# Inhibiting NLRP3 enhances cellular autophagy induced by outer membrane vesicles from *Pseudomonas aeruginosa*

**DOI:** 10.1128/spectrum.01819-24

**Published:** 2025-01-28

**Authors:** Jing Ge, Min Cao, Yuyao Zhang, Tianqi Wu, Jiayi Liu, Jiang Pu, Hongye He, Zhibin Guo, Shaoqing Ju, Juan Yu

**Affiliations:** 1 Department of Laboratory Medicine, Affiliated Hospital of Nantong University 74567 , Nantong, China; 2 Medical School of Nantong University, Nantong, China; 3 Krieger School of Arts and Science, Johns Hopkins University, Baltimore, Maryland, USA; 4 Institute of Public Health, Nantong University, Nantong, China; University of Florida, Gainesville, Florida, USA

**Keywords:** *Pseudomonas aeruginosa*, outer membrane vesicles, autophagy, inflammsome

## Abstract

The bacterium *Pseudomonas aeruginosa* is able to invade lung epithelial cells and survive intracellularly. During this process, it secretes outer membrane vesicles (OMVs), however, it is currently unclear how OMVs from *P. aeruginosa* (PA-OMVs) affect lung epithelial cells and their impact on oxidative stress, autophagy, and other physiological activities of lung epithelial cells. In this study, we found that PA-OMVs activated oxidative stress and autophagy in cells. We demonstrated that the NLRP3 (NLR family, pyrin domain containing 3) inhibitor MCC950 can enhance autophagy induced by PA-OMVs. The main function of NLRP3 is related to the body’s immune response and inflammation regulation. MCC950 is the most common inhibitor of NLRP3. Additionally, we showed that PA-OMVs not only enhanced the expression of AMP-activated protein kinase, a key regulator of cellular energy homeostasis, and reactive oxygen species, which play a crucial role in cellular signaling and oxidative stress, but also significantly enhanced the expression of NLRP3. Inhibiting the expression of NLRP3 further enhanced the process of PA-OMVs induced autophagy. These results demonstrate that PA-OMVs activate both autophagy and the NLRP3 inflammasome, with NLRP3 suppressing autophagy to a certain extent, hoping to provide broad ideas for the future applications of PA-OMVs.

**IMPORTANCE:**

The discovery that lung epithelial cells exposed to outer membrane vesicles from *Pseudomonas aeruginosa* (PA-OMVs) activate cellular autophagy and induce protective immunity is significant. Specifically, the addition of an NLRP3 inhibitor, MCC950, has been found to decrease NLRP3 targets while simultaneously enhancing the autophagy activity induced by PA-OMVs. This finding unveils a novel theoretical framework for the development of PA-OMVs vaccines, highlighting new targets for enhancing the body’s anti-infective responses. By elucidating the mechanisms through which PA-OMVs trigger autophagy and bolster immune defenses, this research opens avenues for innovative vaccine design strategies aimed at combatting infections effectively.

## INTRODUCTION

*Pseudomonas aeruginosa* (PA) is an opportunistic pathogen and drug-resistant bacterium that is being particularly problematic in intensive care units (1). It is a common pathogen in critically ill patients, causing acute or chronic infections in people with immunocompromised illnesses such as cystic fibrosis, sepsis, chronic obstructive pulmonary disease (COPD), and ventilator-associated pneumonia (VAP). It can even colonize the lungs of COPD patients for years or decades (2). Therefore, seeking a novel therapeutic target against PA is a potential way to improve the efficiency.

Bacterial vesicles play an important role in bacterial invasion and reproduction. The characteristics of bacterial vesicles vary according to the characteristics of the parental bacteria. These vesicles are categorized as outer membrane vesicles (OMVs) and membrane vesicles (MVs) based on the gram classification of their parental bacteria (3). Vesicles produced by gram-negative bacteria are termed as OMVs, whereas those produced by gram-positive bacteria do not have an outer membrane in them, and are therefore called MVs (4). A substantial amount of evidence supports the clinical significance of OMVs produced by gram-negative bacteria in driving bacterial pathogenicity and disease in the host (5, 6).

OMVs from *P. aeruginosa* (PA-OMVs) are released when PA infects and colonizes the lung epithelial cells, stimulating the body’s defenses and triggering the initiation of innate immune responses (7, 8); therefore, OMVs are also potential materials for vaccine development (9). Peptidoglycan, lipopolysaccharide (LPS), and other virulence agents carried by OMVs can result in the activation of autophagy (10), leading to the hypothesis that OMVs may also possess autophagy-inducing capabilities. OMVs are naturally occurring, complete structures that encapsulate various bioactive molecules, enabling PA-OMVs to better mimic complex microbial environments and potentially trigger a broader range of physiologically relevant immune responses through multiple signaling pathways. When cells recognize OMVs from *Salmonella*, they activate the AMP-activated protein kinase (AMPK) signaling pathway, which initiates autophagy kinases and promotes xenophagy activation and infection inhibition (11). However, pathogenic strains of *Escherichia coli* can inhibit autophagy to aid bacterial invasion by producing OMVs that exacerbate inflammasome activation (12).

Compared to inactivated PA, PA-OMVs retain intact bioactive molecules, such as LPS, peptidoglycan, and outer membrane proteins, which are crucial for triggering authentic immune responses and simulating real infection scenarios (13). Therefore, PA-OMVs are more suitable for studies on immune activation and autophagy induction, as they provide a more comprehensive and physiologically relevant characterization of bacterial-host cell interactions.

PA is an important opportunistic pathogen in clinical settings, and the study of PA-OMVs has greater clinical relevance (14). Unlike OMVs from *E. coli* and other commensal bacteria, PA-OMVs are more representative of pathogen characteristics in real infection contexts (15). PA-OMVs offer irreplaceable value in research on pathogenic mechanisms and immune regulation, while ensuring that experiments can be conducted under biosafety conditions.

Oxidative stress in cells increases autophagy as well as the activation of NLRP3 inflammasome (16), which plays an important role in the innate immune response triggered by pathogen invasion (17). Autophagy is a self-degradation system that is essential for maintaining cellular homeostasis and serves as a host defense mechanism against infections (18). MCC950, an inhibitor of NLRP3, has demonstrated therapeutic effects in a variety of disorders and studies, including in spontaneous colitis and cholestatic liver injury in mice (19).

In this study, we intend to investigate whether PA-OMVs influence the survival of lung epithelial cells and how OMVs regulate autophagic processes. We concentrated on investigating underlying biological mechanisms, such as signaling pathways related to inflammasomes and autophagy.

## MATERIALS AND METHODS

### Bacterial culture and OMVs isolation

*P. aeruginosa* wild-type strain PAO1 (15692, ATCC) was streaked from −80°C bacterial frozen stock solution (D0391, Beyotime) onto blood agar plates and grown in the presence of 5% CO_2_ overnight at 37°C. After incubation, single colonies were suspended in 1 L LB culture (Sangon Biotech, China), which was shaken at 120 rpm overnight at 37°C until the growth stabilized (OD_600 nm_ >1, viable count of 10^9^ CFU/mL). The culture was subsequently centrifuged at 7,500 rpm for 12 min, and the supernatant was collected, while the precipitate was removed. The supernatant was filtered through a 0.22 µm filter (SLGP033RB, Millipore) and transferred into 15 mL ultrafiltration centrifuge tubes with a 100 kDa cutoff (UFC910024, Millipore). The tubes were centrifuged at 4,000 rpm for 10 min, the filtrate was discarded, and the ultrafiltration membranes were washed with sterile phosphate-buffered saline (PBS). After 2 h of centrifugation at 100,000 × *g* in a sterile ultracentrifuge tube (Beckman, USA), the supernatant was discarded, and the precipitate at the bottom of the tube was resuspended in 1 mL of sterile PBS to extract the OMVs of the appropriate bacterial strains. The extracted PA-OMVs were collected in PBS and kept in a refrigerator at −80℃. The protein concentration of the OMVs was determined using a BCA assay (WB6501, New Cell & Molecular Biotech) (20).

### Analysis of OMVs quantity and size distribution

The NanoSight NS300 (Malvern Instruments, UK) was used to determine the amount and size distribution of OMVs. The size and shape of OMVs were determined using the negative staining approach on a transmission electron microscope. The centrifuged sample was applied dropwise to a carbon-coated copper mesh, negatively stained with 2% hydrogen peroxide acetate. After drying at room temperature, the samples were examined using electron microscopy (21).

### Cell culture and viability assay

Lung epithelial cells A549 (CCL-185, ATCC) were grown in Hams F-12K (F12K) medium (Gibco, USA) supplemented with 10% fetal bovine serum (Sigma, USA) and 1% penicillin/streptomycin (NCM Biotech, Suzhou, China) at 37℃ with 5% CO_2_. Every second day, the medium was renewed until the cells were 80% confluent.

To investigate the cytotoxicity of PA-OMVs, the medium was replaced with an antibiotic-free F12K medium. Trypsin was used to digest A549 cells, followed by centrifugation (1,000 rpm, 5 min) to remove the supernatant. The cells were then diluted to 1 × 10^5^ cells/mL in the same antibiotics-free F12k complete medium.

In total, 100 µL of cell solution was added to each well of 96-well plates and cultured overnight in a 37℃ incubator. After cell adhesion, OMVs were introduced at varying concentrations of 0, 10, 20, 30, 40, and 50 µg/mL. The cells were then incubated at 37℃ for 24 and 48 h to facilitate growth. The CCK-8 assay was used to measure cell viability (C0037, Beyotime). The culture plate was taken out every hour, and the absorbance at 450 nm was read on a microplate reader to calculate the survival rate of the cells. Cell vitality (%) was calculated using the formula: (treated cell absorbance × 100%)/untreated cell absorbance. The cells were cultivated with PA-OMVs at doses of 10, 20, and 30 µg/mL for 2, 4, and 8 h. Unless otherwise stated, the concentration and exposure duration of PA-OMVs in subsequent trials were set at 20 µg/mL for 8 h. To utilize chemical inhibitors, the cells were treated with MCC950 (HY12815, MedChemExpress, 20 µM, 8 h).

### Gene expression analysis by quantitative real-time PCR (qRT-PCR)

Using the TRIzol method (TRIzol reagent, Invitrogen, Carlsbad, CA, USA), total RNA was extracted from the cultures and reverse transcribed into cDNA using a kit in accordance with the manufacturer’s instructions (ThermoFisher Scientific, Waltham, MA, USA). The RNA’s concentration and purity were measured using a UV spectrophotometer (Implant, Munich, Germany). The qRT-PCR was conducted using a LightCycler 480 SYBR Green I Master (Roche, Germany) with a final reaction volume of 20 µL. The expression of target genes was adjusted to that of 18S, a housekeeping gene. The reactions were carried out on a Roche 480 with an initial step of 10 min at 95°C, followed by 40 cycles of 95°C for 15 s and 60°C for 30 s. The primers in this experiment were synthesized by Sangon Biotech (Shanghai, China). Primers were designed with the Primer BLAST algorithm (https://www.ncbi.nlm.nih.gov/tools/primer-blast). The primers are designed as follows, mTOR: F: 5′-GAGATACGCTGTCATCCCTTTA-3′, R: 5′-CTGTATTATTGACGGCATGCTC-3′; LC3B: F: 5′-GATGTCCGACTTATTCGAGAGC-3′, R: 5′-TTGAGCTGTAAGCGCCTTCTA-3′ (22). The others cited from the literature as follows, AMPK: F: 5′-CAACTATCGATCTTGCCAAAGG-3′, R: 5′-AACAGGAGAAGAGTCAAGTGAG-3′ (23); NLRP3: F: 5′-AACAGCCACCTCACTTCCAG-3′, R: 5′-CCAACCACAATCTCCGAATG-3′ (24).

### Reactive oxygen species (ROS) detection

DCFH-DA (S0033S, Beyotime) was diluted in serum-free medium at a ratio of 1:1,000, resulting in a final concentration of 10 µmol/L. After 8 h of PA-OMVs stimulation, the cell culture medium was removed and each well was filled with 1 mL of diluted DCFH-DA solution. The cells were then incubated in a cell culture incubator at 37°C for 20 min. After incubation, to get rid of any DCFH-DA that had not penetrated the cells, the cells were delicately washed three times in media free of serum. Finally, the cells were examined using a fluorescence microscope to access ROS production.

### Western blotting analysis

The A549 cells were lysed in RIPA lysis buffer supplemented with complete protease inhibitor (NCM Biotech, Suzhou, China) after being rinsed with ice-cold PBS solution. Equal amounts of protein (50 µg) were subjected to 12% or 15% sodium dodecyl sulfate-polyacrylamide gel electrophoresis and then transferred to a polyvinylidene difluoride membrane (Beyotime, Shanghai, China) for band separation. Subsequently, the membranes were blocked with 10% skimmed milk, followed by probing with the primary antibodies overnight at 4°C, Beclin 1 (1:1000, YP-AB-00262, UpingBio), LC3B (1:500, A11282, Abclonal), p62 (1:800, WL02385, Wanlebio) and GAPDH (1:10000, AC001, Abclonal) was used. The next day, the blots underwent a thorough cleaning with Tris-buffered saline containing Tween-20 (TBST) before they were incubated with a horseradish peroxidase-conjugated goat anti-rabbit IgG antibody. After 2 h, the blots were thoroughly cleaned with TBST and enhanced chemiluminescence was used to visualize the results.

### Monodansylcadaverine (MDC) staining for autophagy assay

A549 cells were seeded onto six-well plates (1 × 10^4^ cells/mL). After 24 h, the cells were treated with 20 µg/mL of PA-OMVs for 8 h. After incubation, the cells were treated with 50 µM MDC (Solarbio, Beijing, China) for 20 min, followed by Hoechst 33,342 (Beyotime, Shanghai, China) for 5 min (25). Subsequently, fluorescence microscope examination was conducted to capture images.

### Statistical analysis

Each experiment was conducted at least three times, and quantitative data were provided as mean ± S.D. from at least three biological replicates. The data were analyzed using the one-way analysis of variance (ANOVA) with the *post hoc* Tukey test in GraphPad Prism 8.0.2, with a significance level of *P* value < 0.05.

## RESULTS

### Characterization of PA-OMVs

**Fig 1:**
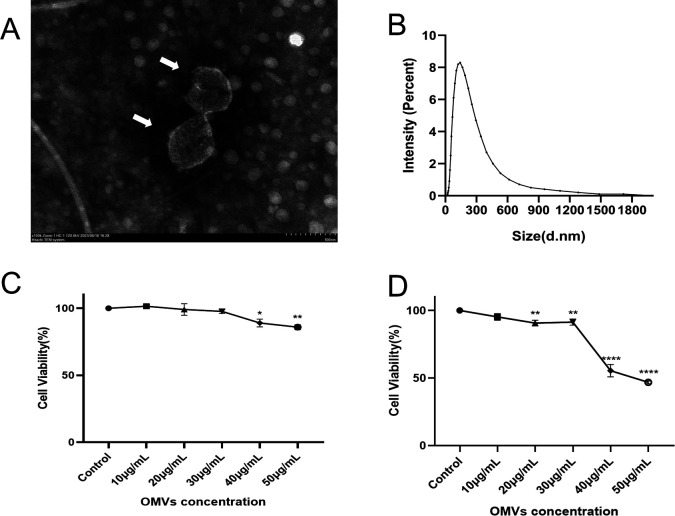
Morphological characterization and cytotoxicity of PA-OMVs. (**A**) PA-OMVs under negative staining electron microscopy. The two arrows point to the two PA-OMVs (*n*  =  3). (**B**) OMVs particle size range was measured by dynamic light scattering analysis (*n*  =  3). (**C**) A549 cell survival after 24 h of OMVs treatment was examined by CCK-8. (**D**) A549 cell survival after 48 h of OMVs treatment was examined by CCK-8. *n*  =  3 independent experiments, one-way ANOVA with *post hoc* Tukey test. *P < 0.05; **P < 0.01; ***P < 0.001; ****P < 0.0001.

The PA-OMVs were purified by ultracentrifugation. To verify the absence of bacteria in the extracted PA-OMVs, bacterial culture experiments were conducted (Fig. S1A and B). In the negative staining electron microscopy field of view, the OMVs appeared as typical round or spherical vesicles encased by lipid bilayers in the shape of “disk cups,” with low electron density components. Malvern particle sizing was used to calculate the approximate particle size of OMVs (Fig. 1A). The particle size varied from 40 to 900 nm, with a peak at 141 nm, indicating that OMVs were effectively purified for use in ensuing cell studies (Fig. 1B). Thus, the approach of obtaining PA-OMVs has been demonstrated to be effective.

### Cytotoxicity of PA-OMVs

Evaluating the potential applications requires assessing the cytotoxicity of PA-OMVs, which was done using the CCK-8 assay. The initial concentration of PA-OMVs was based on the measurements obtained by the BCA assay. A549 cells were treated with various concentrations of PA-OMVs to compare cell survival rates over different durations and concentrations. The results revealed that A549 cells experienced significant damage when treated with PA-OMVs at concentrations above 30 µg/mL for 24 h, while there was no significant cytotoxicity at concentrations of 10 and 20 µg/mL.

Further analysis of cell viability demonstrated that PA-OMVs induced dependence on both concentration and exposure duration. PA-OMVs at concentrations greater than 30 µg/mL had significant cytotoxic effects on cells, and the impact was most prominent after 48 h of exposure (Fig. 1C and D). When cells were treated with 40 and 50 µg/mL of PA-OMVs, cell viability was initially approximately 80% of control cells’ levels, but decreased to 50% as the exposure time increased to 48 h. These findings imply that the safe concentration of PA-OMVs is 30 µg/mL, providing important clues for further study into the toxicity mechanisms of PA-OMVs.

### Oxidative stress in PA-OMVs activated cells

**Fig 2:**
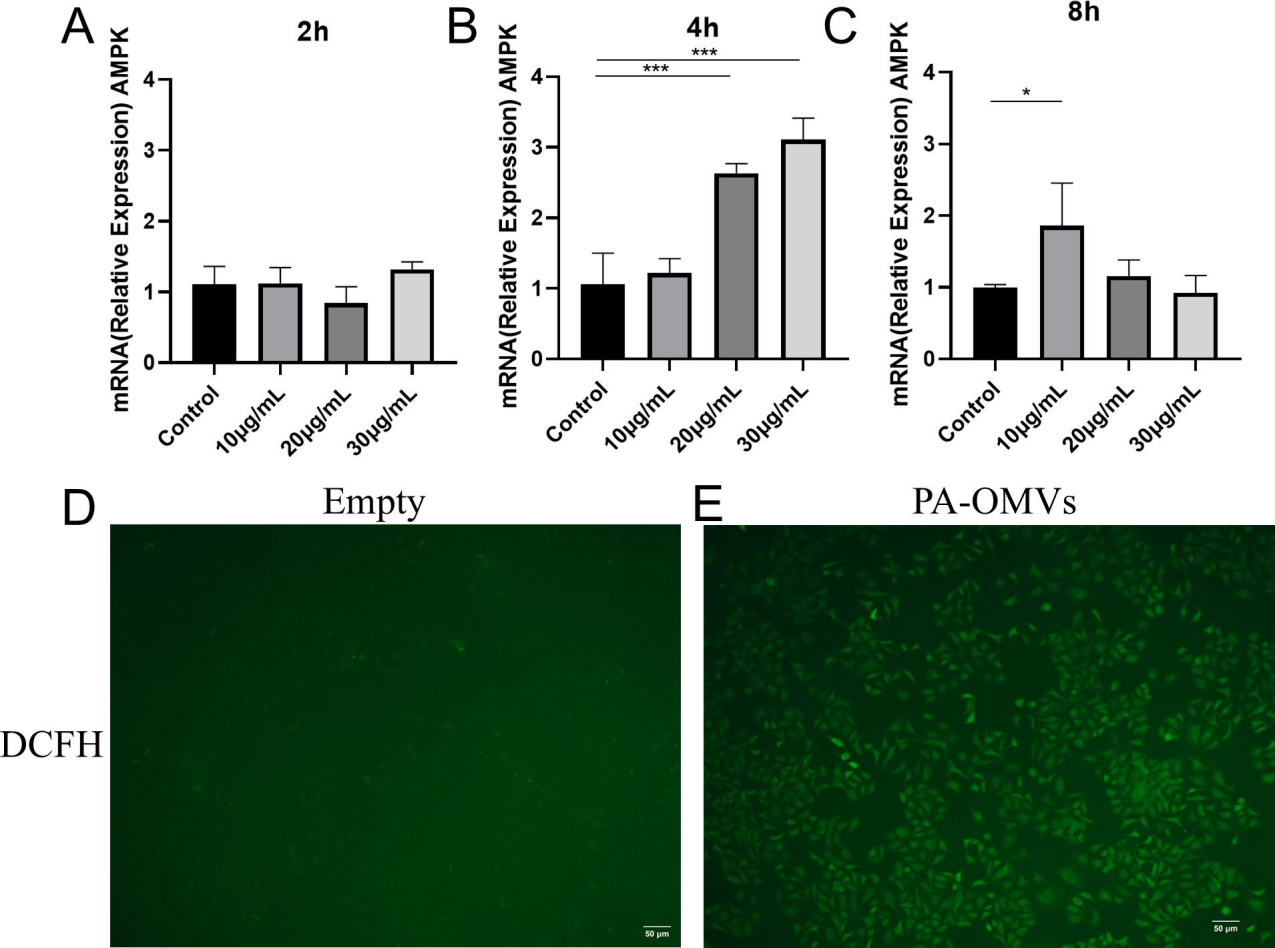
PA-OMVs induced the expression of AMPK and generation of ROS. (**A–C**) qPCR analysis of AMPK expression in A549 cells after 2, 4, and 8 h of OMVs treatment. *n*  =  3 independent experiments, one-way ANOVA with *post hoc* Tukey test. Error bar shows mean ± S.D. (**D and E**) ROS (green) content in Empty group and PA-OMVs treatment group. ROS were detected using the DCFH-DA chemiluminescent probe, and representative images were taken by fluorescence microscopy (*n*  =  3). *P < 0.05; **P < 0.01; ***P < 0.001; ****P < 0.0001.

Prior to the cytotoxic effects exerted by PA-OMVs, oxidative stress is likely triggered in response to cellular stress (26). To determine the preliminary effects of PA-OMVs on cells, we used qRT-PCR to detect the activation of AMPK, a major kinase that maintains energy balance and is implicated in various signaling pathways, including the autophagic pathway (27, 28). After 4 h of treatment with 30 µg/mL PA-OMVs, the expression of AMPK was three times higher than that in the control group. However, after 8 h, the elevation of AMPK expression had diminished, demonstrating that the effect of PA-OMVs on cellular metabolism is phased and did not increase over time (Fig. 2A through C). Additionally, ROS are important particles involved in immune defense (29); fluorescence microscopy was used to detect changes in the fluorescence intensity of the DCFH-DA probe. At the time of peak AMPK expression, ROS levels were measured after 4 h of exposure to 30 µg/mL PA-OMVs. The fluorescence intensity of PA-OMVs-treated A549 cells was significantly greater than that of the control group, indicating that PA-OMVs induced the production of ROS (Fig. 2D and E).

### PA-OMVs promote autophagy in A549 cells

**Fig 3:**
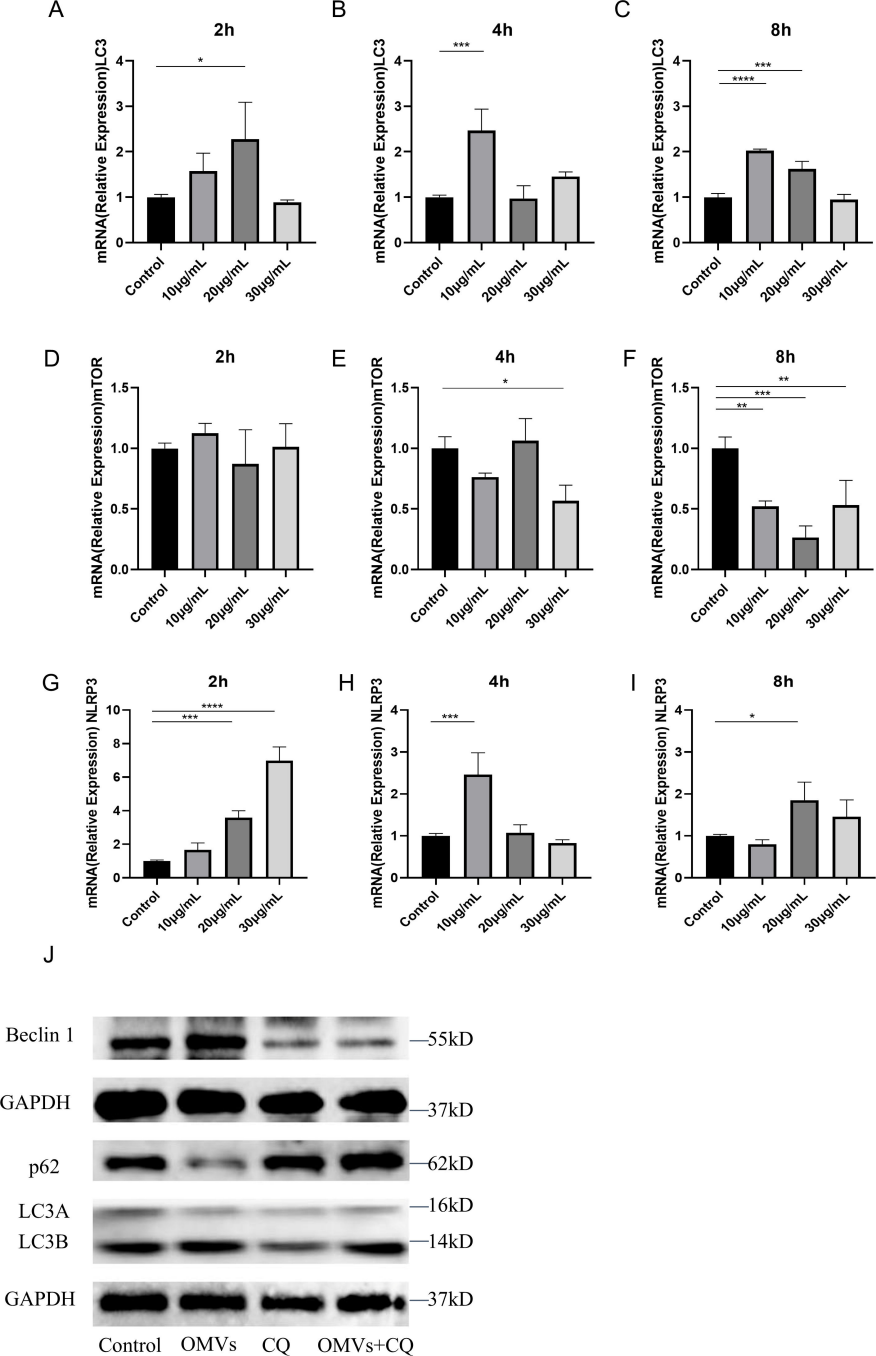
PA-OMVs promoted autophagy in A549 cells. (**A–C**) Expression of LC3 in A549 cells after 2, 4, and 8 h of OMVs treatment was quantified using qPCR (*n*  =  3). (**D–F**) Expression of mTOR in A549 cells after 2, 4, and 8 h of OMVs treatment was quantified using qPCR (*n*  =  3). mTOR inhibits autophagy by suppressing key autophagic pathways, and at a concentration of 20 µg/mL with an exposure time of 8 h, mTOR expression reaches its lowest level. (**G–I**) Expression of NLRP3 in A549 cells after 2, 4, and 8 h of OMVs treatment was quantified using qPCR (*n* = 3). Data are mean ± S.D, one-way ANOVA with *post hoc* Tukey test. (**J**) p62, Beclin 1, LC3B/LC3A in A549 cells after 8 h of OMVs treatment protein expression (*n* = 4). (CQ: the autophagy inhibitor chloroquine). Western blots showing that the addition of OMVs promoted an increase in Beclin 1, an early autophagy protein involved in autophagosome initiation, a decrease in p62, an autophagy substrate whose reduction indicates enhanced autophagic flux, and an elevated LC3B/LC3A ratio, which reflects increased autophagosome formation and activity. Conversely, treatment with an autophagy inhibitor resulted in a decrease in Beclin 1, an increase in p62, and a reduced LC3B/LC3A ratio, indicating that PA-OMVs can induce autophagy in A549 cells. PA-OMVs can promote autophagy in A549 cells, as evidenced by the inhibitor CQ. *P < 0.05; **P < 0.01; ***P < 0.001; ****P < 0.0001.

Autophagy is a natural immune system defense against harmful bacterial infections (30). The activation of autophagy means the triggering of the body’s defense mechanism against OMVs (26). To explore whether PA-OMVs stimulate autophagy, we analyzed autophagy-associated mRNA expression (LC3B, mTOR) and the inflammasome-associated molecule NLRP3 in PA-OMVs exposed to A549 cells at different time intervals. The results showed that LC3B mRNA expression increased significantly after treatment with 20 µg/mL of PA-OMVs at 2 h (Fig. 3A through C). In contrast, mTOR mRNA expression remained largely unchanged at 2 and 4 h, but showed a significant decrease at 8 h, highlighting its role as a negative feedback regulator (Fig. 3D through F). However, autophagy is a dynamic process at the protein level, and mRNA detection is only a preliminary determination. For NLRP3, its mRNA expression was modestly elevated at 2 and 4 h, but the overall trend is stable. Whereas at 8 h, a notable increase in NLRP3 mRNA expression was observed compared to the control (Fig. 3G through I). The condition of 8 h at 20 µg/mL of PA-OMVs, which yielded the highest mRNA expression of NLRP3, was selected for the subsequent measurement of protein expression (LC3B/LC3A, p62, and Beclin 1). In the process of autophagy, various cellular components are captured by autophagosomes and then eliminated after merging with lysosomes (31). Autophagy is defined by the presence of microtubule-associated protein 1 light chain 3 (LC3), and its variants LC3B/LC3A serve as markers of autophagy occurrence (32). Beclin 1 is essential for the initial phase of autophagy (33), and p62, when autophagy is active, p62 is degraded, reducing its levels. Conversely, impaired autophagy causes p62 to accumulate, making it a key indicator of autophagic activity (34).

The Western blotting results indicated an increase in LC3B/LC3A expression, a decrease in p62 expression, and a dramatic increase in Beclin 1 expression in cells exposed to PA-OMVs. To eliminate interference, the autophagy inhibitor chloroquine (CQ) was utilized to see if comparable results would emerge (35). In the presence of CQ (20 µmol/L), the OMVs-treated group exhibited significantly lower levels of autophagy compared to the control group. This was evidenced by higher levels of p62 and LC3B/LC3A, indicating that CQ effectively blocked the autophagic activities stimulated by PA-OMVs. Furthermore, the ratio of LC3B/LC3A and Beclin 1 in PA-OMVs exposed cells was significantly higher than in the control, indicating an enhanced autophagy level. These results confirm that PA-OMVs can induce autophagy in A549 cells (Fig. 3J). The band intensity graphs are shown in Fig. S2A through C.

### NLRP3 inhibitor MCC950 enhanced autophagy induced by PA-OMVs

**Fig 4:**
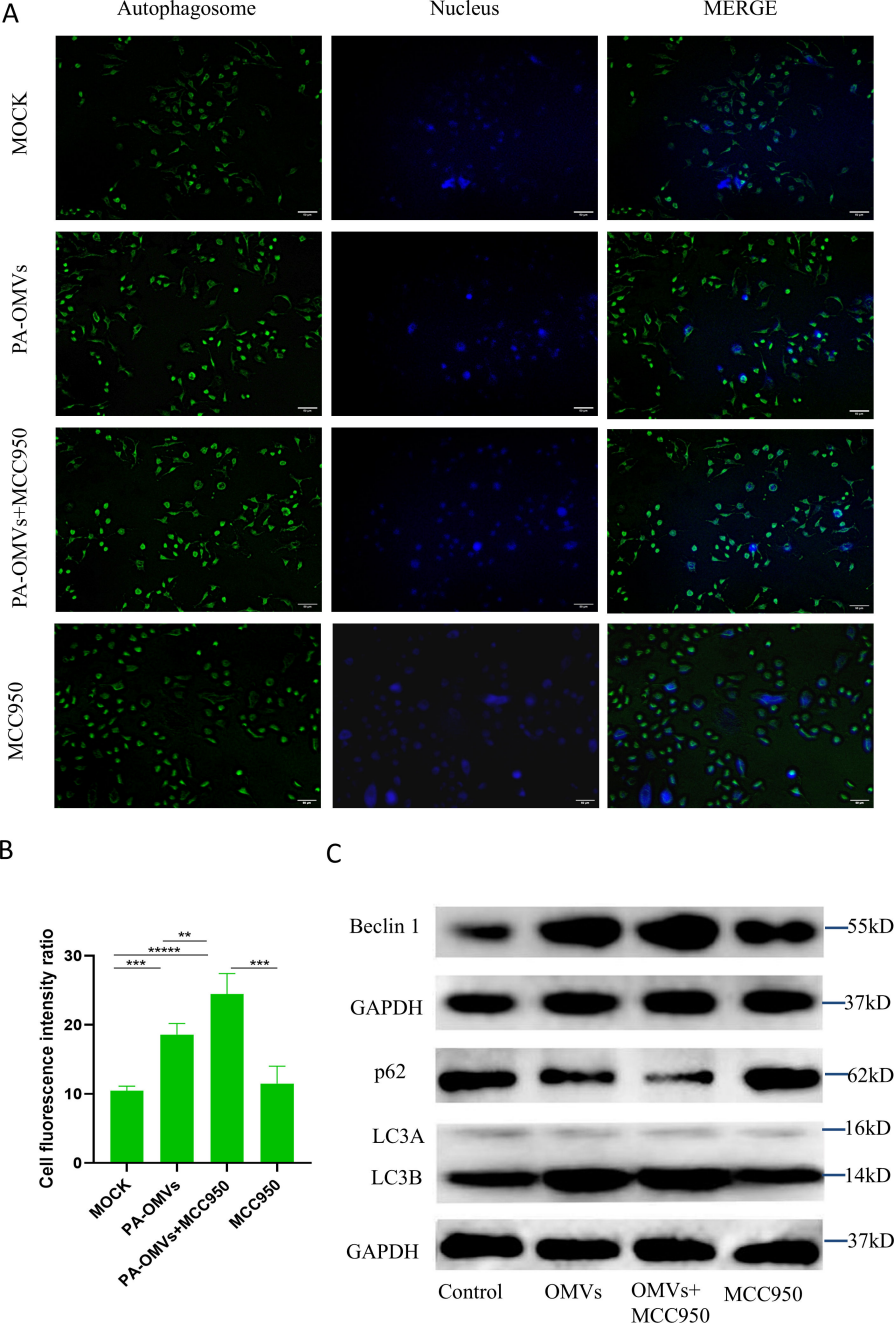
NLRP3 inhibitor MCC950 enhanced autophagy induced by PA-OMVs. (**A**) Number of autophagic vesicles in A549 cells after 8 h of OMVs treatment as observed by MDC staining. The nucleus was stained with Hoechst 33342 (blue) and autophagosome was stained with MDC (green) (*n*  =  3). (**B**) Quantification of autophagosome based on microscopic images in A549 cells after 8 h of 20 µg/mL OMVs treatment. Data are mean ± S.D, one-way ANOVA with *post hoc* Tukey test. (**C**) Western blots showing that the combination of OMVs and MCC950 promoted an increase in Beclin 1, a key early autophagy protein involved in autophagosome initiation, a decrease in p62, an autophagy substrate whose reduction indicates enhanced autophagic flux, and an elevated LC3B/LC3A ratio, a marker of autophagosome activity. In the presence of MCC950 alone, Beclin 1 showed a slight increase compared to the Control group but remained lower than in the PA-OMVs + MCC950 group. Meanwhile, p62 and the LC3B/LC3A ratio showed no significant changes. These results indicate that MCC950 enhances PA-OMVs-induced autophagy (*n*  =  4). *P < 0.05; **P < 0.01; ***P < 0.001; ****P < 0.0001.

Considering the enhancement of NLRP3 by PA-OMVs in the previous experiments, MCC950, an NLRP3 inhibitor, was introduced in response to preliminary findings. In this study, we found that PA-OMVs had a substantial effect on the expression of the inflammasome molecule NLRP3. To investigate whether MCC950 enhanced autophagy in response to PA-OMVs *in vitro*, autophagosome formation was examined through MDC staining. The intracellular intense bright green fluorescence observed in the MDC staining image indicated the formation of autophagosomes in A549 cell cytoplasm (Fig. 4A). In contrast, the control cells displayed slight green fluorescence. PA-OMVs triggered autophagy, resulting in much stronger green fluorescence in the cytoplasm of cells treated with both PA-OMVs and MCC950. Subsequently, the intensity of green fluorescence in each set of A549 cells was measured using ImageJ software. The fluorescence intensity of autophagic vesicles was much higher in the PA-OMVs group compared to the control group, suggesting PA-OMVs' ability to activate autophagy in lung epithelial cells. Furthermore, with MCC950 incubation, the PA-OMVs + MCC950 group triggered more autophagy in A549 cells (Fig. 4B).

It was further verified at the protein level that MCC950 exhibits a specific enhancing effect on autophagy induced by PA-OMVs. In the OMVs + MCC950 group, the expression of Beclin 1 in cells in the OMVs + MCC950 group was enhanced. It was also observed that when MCC950 was used alone, Beclin 1 levels increased slightly. Since Beclin 1 is an autophagy-initiating protein, inhibiting NLRP3 under normal conditions may also trigger Beclin 1. The expression of LC3B/LC3A was significantly increased compared to the PA-OMVs group, while the expression of p62 was significantly decreased. Therefore, we concluded that inhibition of the NLRP3 target by MCC950 made the autophagy-promoting effect of PA-OMVs more effective, facilitating the conversion of LC3A to LC3B and promoting the degradation of p62 (Fig. 4C). The band intensity graphs are shown in Fig. S2D through F.

## DISCUSSION

Autophagy serves as effective waste degradation system that helps organisms maintain a reasonably steady homeostatic state (36). Furthermore, autophagy assists the host in combating bacterial infections (37). Studies report that autophagy shields against Group A streptococcus infections (38). To avoid pathogens, the cells use autophagy to fight infections and maintain the body’s balance. Some bacteria have been shown to impede autophagy by interfering with the production of autophagosomes. OMVs can serve as a bacterial zero secretion system (39), crucial in delivering virulence factors, such as nucleic acids, proteins, peptidoglycan, and LPS. When OMVs enter the host’s cytoplasmic lysate, OMVs prompt an inflammatory immune response by activating noncanonical inflammasome machinery through LPS detection (7). In many studies, LPS or peptidoglycan are considered key components in activating autophagy (40). However, in PA-OMVs, LPS and peptidoglycan are only part of the whole structure. In this study, as a complete biological entity, OMVs can interact with host cells to trigger a range of biological responses, including oxidative stress, activation of the NLRP3 inflammasome, and regulation of the autophagy process. Investigating PA-OMVs as a whole allows for a comprehensive understanding of their role in cellular autophagy.

There are many studies examining the impact of OMVs on cellular autophagy, though the effects of OMVs produced by different bacteria vary. OMVs produced by certain *E. coli*, for example, effectively eliminate colorectal cancer cells (41). However, OMVs can also be used as tools to inhibit autophagy. It has been reported that OMVs produced by HlyF-expressing pathogenic *E. coli* cause macrophage IL-1β release and extensive cell death (12). Our research indicates that this overactivation is due to OMVs interfering with autophagy and disrupting the mechanisms that normally provide negative feedback. In some ways, autophagy resembles a true form of immunity. Based on these results, it was speculated that PA-OMVs may play a role in autophagy induction. Currently, there are no relevant studies on how PA-OMVs increase autophagy in lung epithelial cells and whether MCC950 activates autophagy by inhibiting NLRP3.

In our study, PA-OMVs had a significant effect on cell survival. A high concentration of PA-OMVs caused obvious cytotoxicity. Low concentrations of PA-OMVs can increase the expression of a major kinase that maintains energy balance, AMPK, increase the production of intracellular ROS, and activate oxidative stress in lung epithelial cells. The increase in ROS implies that PA-OMVs induce oxidative stress within cells. ROS have been widely reported as early inducers of autophagy (42) and can activate the NLRP3 inflammasome (16, 43, 44). NLRP3 is an inflammasome sensor protein that senses many pathogens and is associated with the occurrence and development of autoinflammatory and autoimmune diseases (45, 46). For example, OMVs from *E. coli* can cause mitochondrial apoptosis in macrophages and activate the NLRP3 inflammasome (47).

MCC950 is a renowned inhibitor that blocks the NLRP3 target, but knowledge of its role in autophagy remains poorly understood. There is currently strong evidence suggesting a link between autophagy and NLRP3 inflammasome, with these two processes influencing and affecting each other (48). On the one hand, the activation of NLRP3 inflammasome may promote autophagy (49), whereas excessive autophagy may be reduced by inflammasome activation (36).

Our findings indicate that exposure of lung epithelial cells to PA-OMVs can activate autophagy and elicit protective immunity. Adding the NLRP3 inhibitor MCC950 can inhibit the NLRP3 inflammasome and increase the activation of autophagy by PA-OMVs. Inhibiting the NLRP3 inflammasome may lead to enhanced autophagy, implying the existence of a negative regulatory mechanism between these processes. This negative feedback likely results from the overlapping pathways between PA-OMVs' activation of NLRP3 inflammasome and autophagy, and inhibition of one pathway may lead to compensatory activation of the other pathway. The research on these aspects deserves further exploration. Our investigation on PA-OMVs autophagy provides a theoretical foundation for the later development of PA-OMV vaccines and the identification of new anti-infection targets. Autophagy induction by MCC950 could potentially serve as an effective adjuvant for future PA-OMV vaccines.

## Data Availability

All data generated or analyzed during this study are included in this published article and in the supplementary material of this article.

## SUPPLEMENTAL MATERIAL

Supplemental figures spectrum.01819-24-s0001.docx Fig. S1 and S2.

ASM does not own the copyrights to Supplemental Material that may be linked to, or accessed through, an article. The authors have granted ASM a non-exclusive, world-wide license to publish the Supplemental Material files. Please contact the corresponding author directly for reuse.
