# Supplementary material for: Inhibiting NLRP3 enhances cellular autophagy induced by outer membrane vesicles from Pseudomonas aeruginosa
Source: Microbiol Spectr. 2025 Jan 28;13(3):e01819-24. doi: 10.1128/spectrum.01819-24 (PMC11878092; doi:10.1128/spectrum.01819-24)
Supplement: Supplemental figures — Fig. S1 and S2. [file spectrum.01819-24-s0001.docx]

Supplemental Figure S1：OMVs bacterial culture experiments.


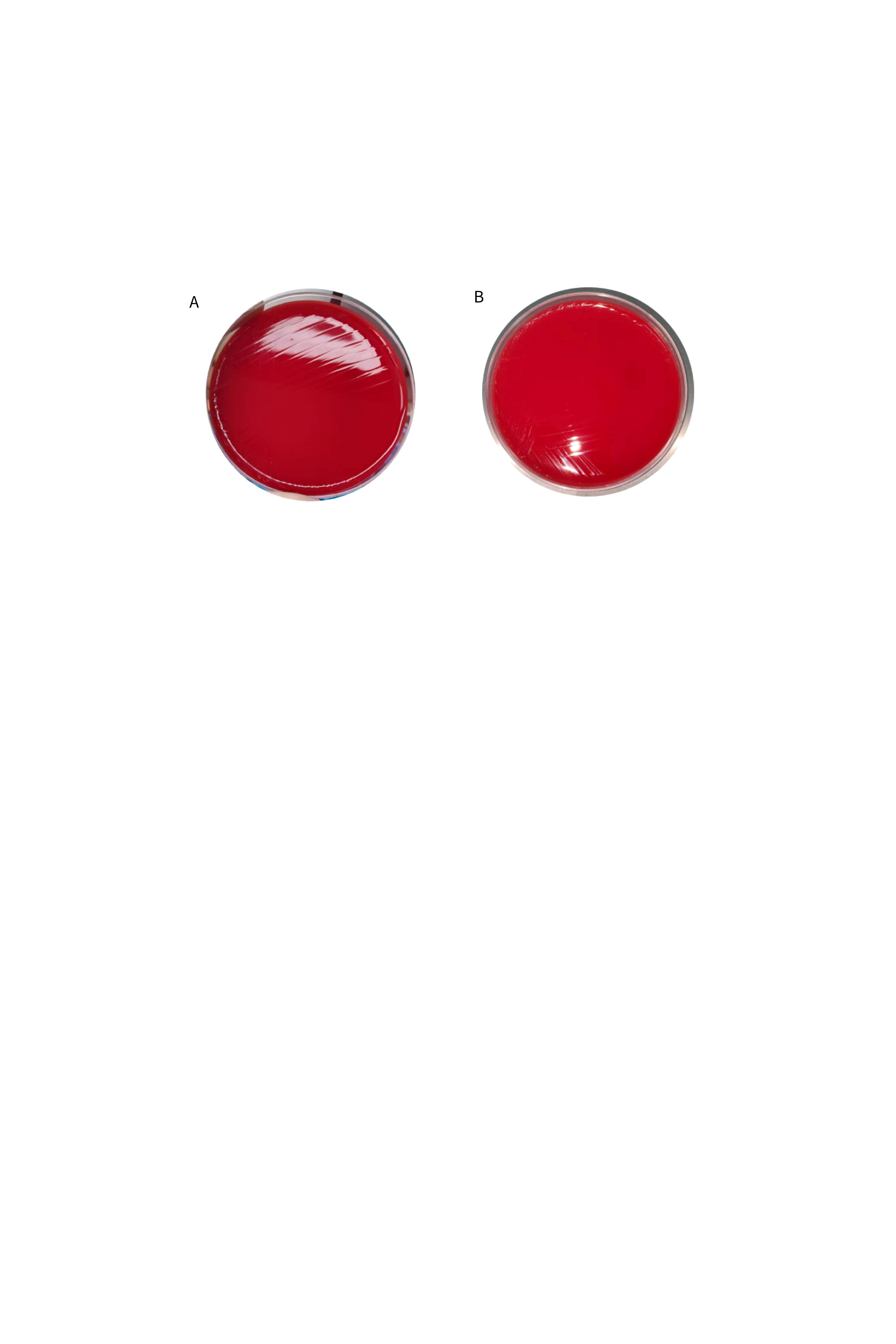


1. OMVs were cultured for 24h. (B) OMVs were cultured for 48h.

Supplemental Figure S2: The band intensity graphs of Western blots.


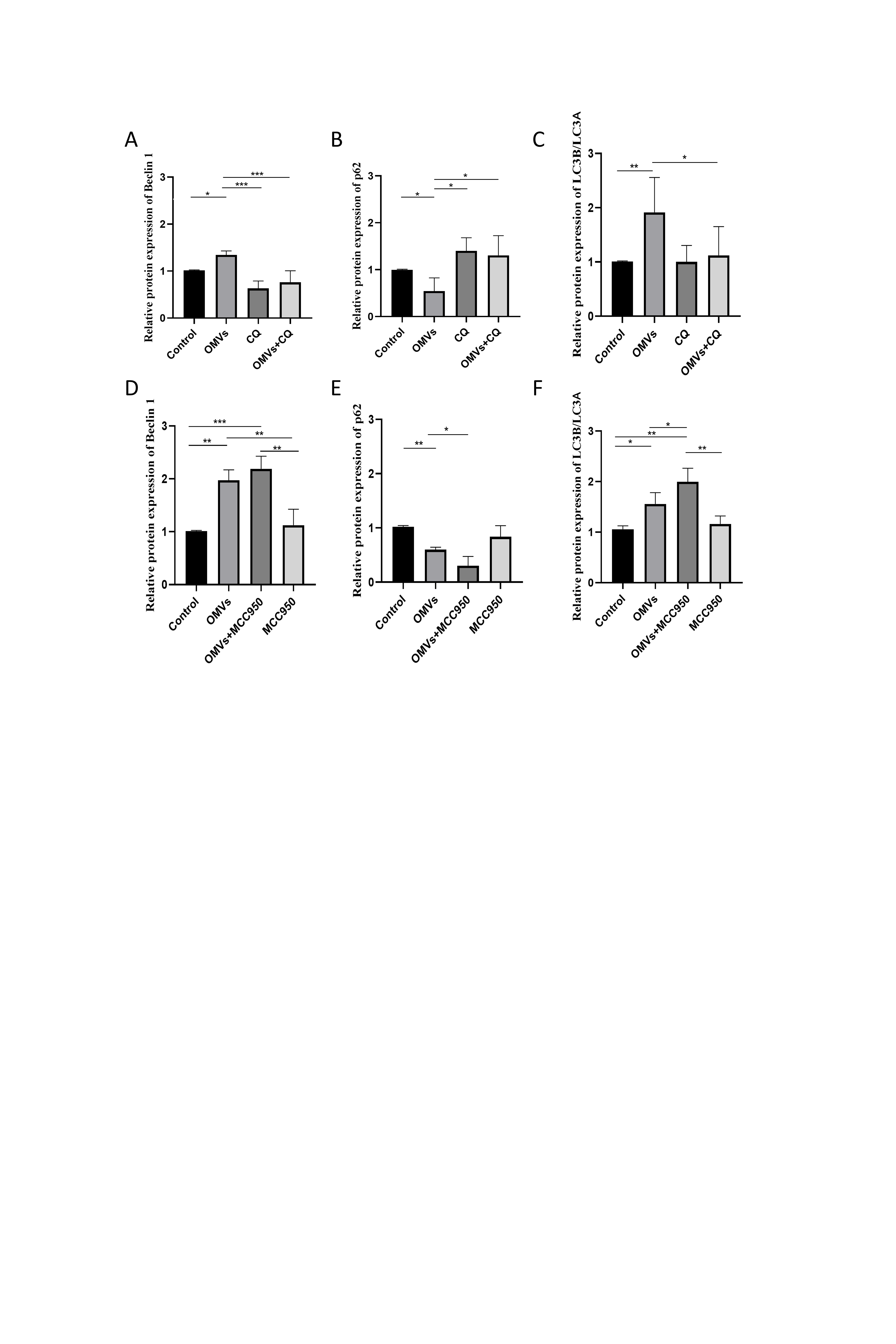


1. C) Quantification of p62, Beclin 1, LC3B/LC3A in A549 cells treated with PA-OMVs by Western blotting. (D-F) Quantification of p62, Beclin 1, LC3B/LC3A in A549 cells treated with PA-OMVs and NLRP3 inhibitor MCC950 by Western blotting. n = 4 independent experiments, one-way ANOVA with posthoc Tukey test. *，*P* <0.05; **，*P*<0.01; ***，*P*<0.001； ****，*P*<0.0001.
